# Efficacy of intranasal LaAg vaccine against *Leishmania amazonensis* infection in partially resistant C57Bl/6 mice

**DOI:** 10.1186/s13071-016-1822-9

**Published:** 2016-10-06

**Authors:** Juliana Elena Silveira Pratti, Tadeu Diniz Ramos, Joyce Carvalho Pereira, Alessandra Marcia da Fonseca-Martins, Diogo Maciel-Oliveira, Gabriel Oliveira-Silva, Mirian França de Mello, Suzana Passos Chaves, Daniel Claudio Oliveira Gomes, Bruno Lourenço Diaz, Bartira Rossi-Bergmann, Herbert Leonel de Matos Guedes

**Affiliations:** 1 Laboratório de Inflamação, Instituto de Biofísica Carlos Chagas Filho, Universidade Federal do Rio de Janeiro, Rio de Janeiro, RJ Brazil; 2 Núcleo Multidisciplinar de Pesquisa UFRJ–Xerém em Biologia (NUMPEX-BIO), Polo Avançado de Xerém–Universidade Federal do Rio de Janeiro, Duque de Caxias, Rio de Janeiro Brazil; 3 Laboratório Integrado de Imunoparasitologia, Campus Macaé–Universidade Federal do Rio de Janeiro, Macaé, Brazil; 4 Laboratório de Imunobiologia, Núcleo de Doenças Infecciosas/Núcleo de Biotecnologia, Universidade Federal do Espírito Santo, Vitória, ES Brazil; 5 Laboratório de Imunofarmacologia, Instituto de Biofísica Carlos Chagas Filho, Universidade Federal do Rio de Janeiro, Rio de Janeiro, RJ Brazil

**Keywords:** Leishmaniasis, *Leishmania amazonensis*, Intranasal vaccine, LaAg, C57BL/6, ADDAVAX*®*

## Abstract

**Background:**

We have previously demonstrated that intranasal vaccination of highly susceptible BALB/c mice with whole *Leishmania amazonensis* antigens (LaAg) leads to protection against murine cutaneous leishmaniasis. Here, we evaluate the response of partially resistant C57BL/6 mice to vaccination as a more representative experimental model of human cutaneous leishmaniasis.

**Methods:**

C57BL/6 mice from different animal facilities were infected with *L. amazonensis* (Josefa strain) to establish the profile of infection. Intranasal vaccination was performed before the infection challenge with two doses of 10 μg of LaAg alone or associated with the adjuvant ADDAVAX® by instillation in the nostrils. The lesion progression was measured with a dial caliper and the parasite load by limited dilution assay in the acute and chronic phases of infection. Cytokines were quantified by ELISA in the homogenates of infected footpads.

**Results:**

C57BL/6 mice from different animal facilities presented the same *L. amazonensis* infection profile, displaying a progressive acute phase followed by a controlled chronic phase. Parasites cultured in M199 and Schneider’s media were equally infective. Intranasal vaccination with LaAg led to milder acute and chronic phases of the disease. The mechanism of protection was associated with increased production of IFN-gamma in the infected tissue as measured in the acute phase. Association with the ADDAVAX® adjuvant did not improve the efficacy of intranasal LaAg vaccination. Rather, ADDAVAX® reduced vaccination efficacy.

**Conclusion:**

This study demonstrates that the efficacy of adjuvant-free intranasal vaccination with LaAg is extendable to the more resistant C57Bl/6 mouse model of infection with *L. amazonensis*, and is thus not exclusive to the susceptible BALB/c model. These results imply that mucosal immunomodulation by LaAg leads to peripheral protection irrespective of the genetic background of the host.

**Electronic supplementary material:**

The online version of this article (doi:10.1186/s13071-016-1822-9) contains supplementary material, which is available to authorized users.

## Background

*Leishmania amazonensis* is a causative agent of localized and diffuse cutaneous leishmaniasis in Latin America [1, 2]. In Brazil, infections with *L. amazonensis* used to be concentrated in the North of the country (Amazon Forest Region) [3]. In Manaus, 8 % of cutaneous infections were caused by *L. amazonensis* [4]. Since 2005, the Brazilian Ministry of Health has demonstrated the presence of *L. amazonensis* in all regions of Brazil [3]. The concern about *L. amazonensis* in Brazil relates to all forms of disease, including visceral and mucosal leishmaniasis [5] and the refractoriness to treatment of serious forms of the infection [6]. Difficulty in access to the regions affected by the disease hinders treatment efforts [3], thus the best strategy is prevention through vaccination.

*Leishmania amazonensis* is highly virulent with capacity to infect several hosts [7]. BALB/c mice have been used for several studies; however, this model of infection is a progressive non-healing disease. This fate is not related to the most prevalent presentation of natural cutaneous infection in human populations, which is characterized by an open spontaneously healing wound, leaving an unpleasant scar containing parasites [7]. C57BL/10 mice present the same phenotype as BALB/c after experimental infection with *L. amazonensis* [8]. However, in C57BL/6 mice, the infection was described to have a distinctive progressive [9] and a non-progressive disease profile [10] even for the same parasite strain (MHOM/BR/77/LTB0016). Some differences in in vivo infection could be associated to differences in strains [11], time post-infection studied, challenge used, site of infection and infection route used [12]. Furthermore, the differences in microbiota is currently known to affect the immune response in mice of the same background [13, 14].

The development of a vaccine against different *Leishmania* parasites is the priority to control leishmaniasis [15]. Unfortunately, we do not have any vaccine approved for human use [16]. The Leishvacin® (or LaAg) vaccine, comprised of whole *Leishmania amazonensis* antigens, has been studied for several years. Although the safety and capacity to induce IFN-gamma production was demonstrated [17], the vaccine failed in the phase 3 of a clinical trial [18]. It is noteworthy that these trials were performed using the subcutaneous or intramuscular route of administration. Using experimental models and the same route, the immunization with LaAg in monkeys [19] or BALB/c mice [20] exacerbated the disease progression of *L. amazonensis* infection. However, when the same antigen was tested by intranasal route, it induced protection on BALB/c mice [21]. Mucosal vaccine elicits immune responses effective against several pathogens [22], and the intranasal route has been effective against leishmaniasis using BALB/c mice [23–28] and hamster [29, 30] models.

To improve vaccine efficacy, several adjuvants have been studied for use by the mucosal route [26, 28, 31]. Protective responses of Leish111f [26] and recombinant LACK [28] were improved when associated to cholera toxin, but this adjuvant is not approved for human use [31]. The only adjuvant approved for intranasal use is the MF59® [32]. A similar adjuvant called ADDAVAX®, a nano oil-water emulsion formulated with scalene, was developed by Invitrogen. Intranasal LaAg vaccine is effective without association of adjuvants against leishmaniasis [21] and the association with adjuvants, as ADDAVAX®, could enhance the protective immunity.

In this paper, we established the infection model of C57BL/6 from different animal facilities using *L. amazonensis* (strain MHOM/BR/75/Josefa). This strain was isolated from a patient with cutaneous leishmaniasis (the most common form of the disease) in 1975 by Dr. Cesar Cuba-Cuba (Universidade de Brasília, Brasília, Brazil). We evaluated the LaAg intranasal vaccine in this mouse model. The intranasal LaAg vaccine induced partial protection during the progressive and chronic phase against *L. amazonensis* on C57BL/6.

## Methods

### Animals

C57BL/6 mice were acquired from different animal breeding facilities: Universidade Federal Fluminense (C57Bl/6-UFF), Universidade Federal do Rio de Janeiro (C57Bl/6-UFRJ), Fundação Oswaldo Cruz (C57Bl/6-FIOCRUZ) and Universidade Estadual de Campinas (C57Bl/6-UNICAMP). BALB/c mice were from UFF animal facility. Animals were maintained in our own animal facility at UFRJ using sterilized bedding, filtered water and pelleted food. For experiments, females were used at 6–8 weeks of age.

### Parasites

For infection experiments, *L. amazonensis* (strain MHOM/BR/75/Josefa) [33] and *L. amazonensis* (MPRO/BR/72/M1845, LV78 strain) [34] promastigotes were maintained at 26 °C in M199 medium containing 10 % heat-inactivated fetal bovine serum (HIFCS, GIBCO Laboratories, Grand Island, NY, USA) or Schneider’s medium containing 10 % HIFCS until the stationary-growth phase. The Josefa strain was originally isolated from cutaneous leishmaniasis [33], whereas the LV78 strain was isolated from skin of the rat *Proechimis* sp. [34]. Quantification of metacyclic promastigotes was performed routinely and was around 50 % using Ficoll density gradient*.*

### LaAg preparation

*Leishmania amazonensis* (MHOM/BR/75/Josefa strain) promastigotes were maintained at 26 °C in M199 medium containing 10 % HIFCS. *Leishmania amazonensis* promastigote antigens (LaAg) were prepared as previously described [35]. Briefly, stationary-growth phase promastigotes were washed three times in phosphate buffered saline (PBS) and subjected to three cycles of freezing and thawing. LaAg was lyophilized, stored at -20 °C and reconstituted with PBS immediately prior to use.

### Immunization, infection challenge and evaluation of disease progression

Mouse immunization was by instillation of 10 μg of LaAg in 20 μl of PBS, 10 μl in each nostril, using a micropipette adapted with a polystyrene microtip. A booster dose was given 7 days later [21]. Controls received PBS alone. For association with adjuvant, 10 μg of LaAg (in 10 μl) was mixed by pipetting with 10 μl of ADDAVAX®, and 10 μl were administered in each nostril. Seven days post-boost, animals were infected in the right hind footpad with 5 × 10^5^ or 2 × 10^6^ stationary-phase *L. amazonensis* promastigotes. Lesion sizes were measured once a week with a dial caliper and expressed as the difference between the thicknesses of infected and contralateral non-infected footpads. The parasite load was determined at the end of the experiments, when the infected foot was skinned and individually homogenized in 1 ml of PBS using a tissue grinder. Tissue debris was removed by gravity sedimentation for 5 min. Homogenates were submitted to limited dilution assay (LDA).

### Cytokine quantification

For *in situ* production [24], infected footpads were isolated, skinned, weighed, teased and individually homogenized in 1 ml of PBS using a glass tissue homogenizer. The footpad homogenates were centrifuged (10 min, 20,000 × *g* at 4 °C) and the supernatants collected. For cytokine quantification, supernatants prepared as above were assayed for TGF-β, IFN-γ, IL-10 and IL-4 by ELISA following the manufacturer’s instructions (R&D Systems, Minneapolis, USA). For TGF-β, the supernatants were pre-heated to 80 °C for 5 min prior to the assay.

### Flow cytometry

Lymph node cells isolated from mice were cultured for 4 h to at 37 °C in the presence of PMA (20 ng/ml), Ionomycin (1μg/ml) and brefeldin A (Sigma-Aldrich, St. Lois, USA). Cells were surface stained with Anti-CD3-Percp and anti-CD8-FITC and anti-CD4-PE CY7 (Biolegend, San Diego, USA) and fixed and permeabilized for 1 h using Foxp3/Transcription Factor Fixation/Permeabilization Kit (e-Bioscience, Santa Clara, USA). Intracellular cytokine staining was performed with anti- IFN-γ -APC (Biolegend). At least 10,000 gated CD4^+^ lymphocyte events were acquired. Analytical flow cytometry was conducted with a BD FACSCanto™ II (BD Biosciences New Jersey, USA) and the data were processed with FlowJo X software.

### Statistical analysis

**Fig. 1:**
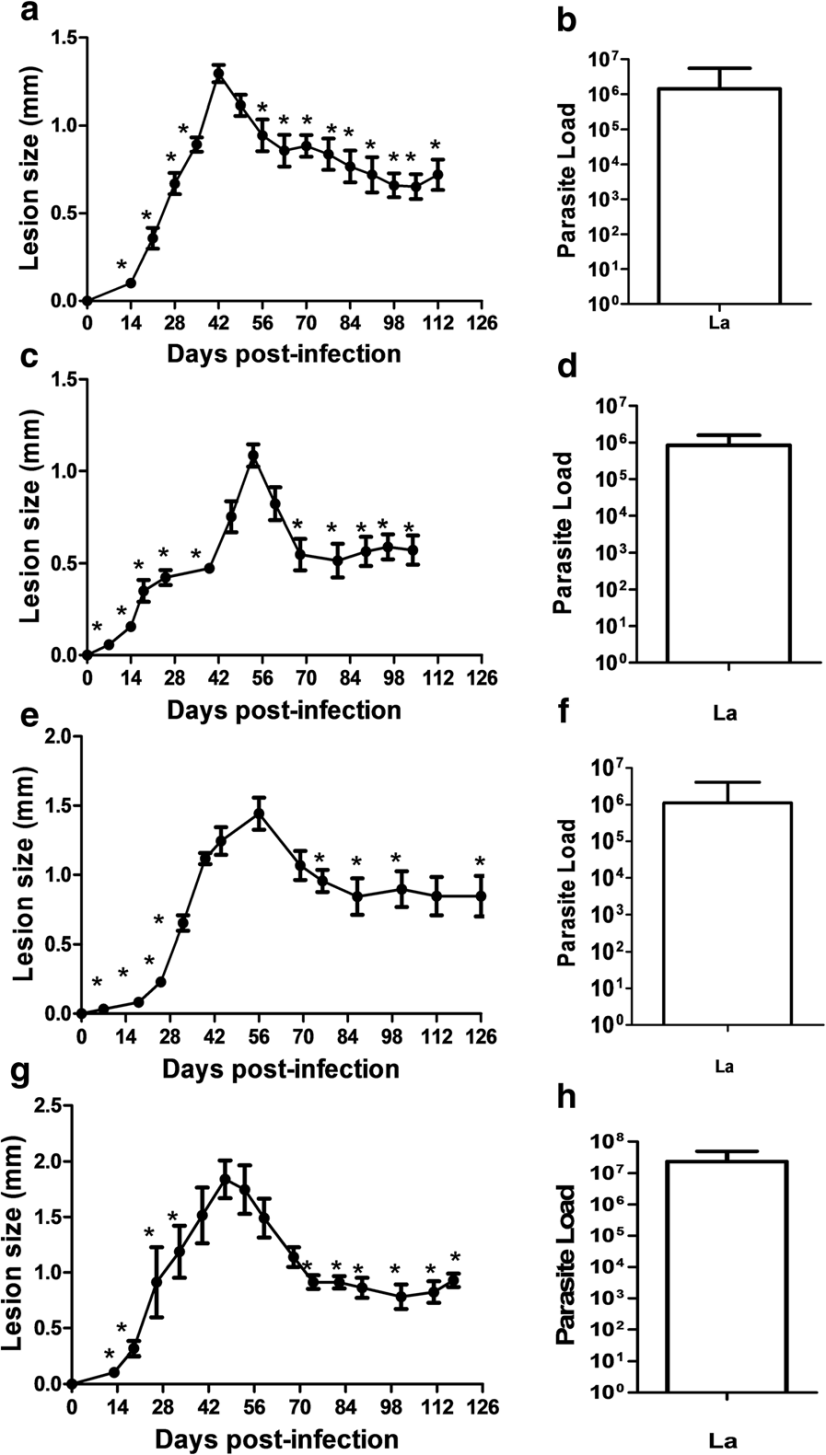
Course of infection by *L. amazonensis* challenge (Josefa strain) in C57BL/6 mice from different sources. *Leishmania amazonensis* were cultured on M199 Medium. C57Bl/6-UNICAMP (**a**, **b**), C57Bl/6-FIOCRUZ (**c**, **d**), C57Bl/6-UFRJ (**e**, **f**) and C57Bl/6-UFF (**g**, **h**) were infected in the footpads with 5 × 10^5^ stationary-phase promastigotes of *L. amazonensis* by subcutaneous route. Lesion sizes were measured at the indicated days and are expressed as the difference in thickness between non-infected and infected footpads (**a**, **c**, **e**, **g**). Parasite load was measured at the end of the experiment and expressed as the mean number of parasites in each footpad (**b**, **d**, **f**, **h**). The data (means ± standard deviations; *n* = 4–5) are representative of two (**a**, **b**) and three (**c**, **d**, **e**, **f**, **g**, **h**) independent experiments producing the same result profile. *P ≤ 0.05 in comparison to peak of infection (**a**, 42 days; **c**, 56 days; **e**, 56 days, **g**, 53 days; see Table 1 for details)

**Fig. 2:**
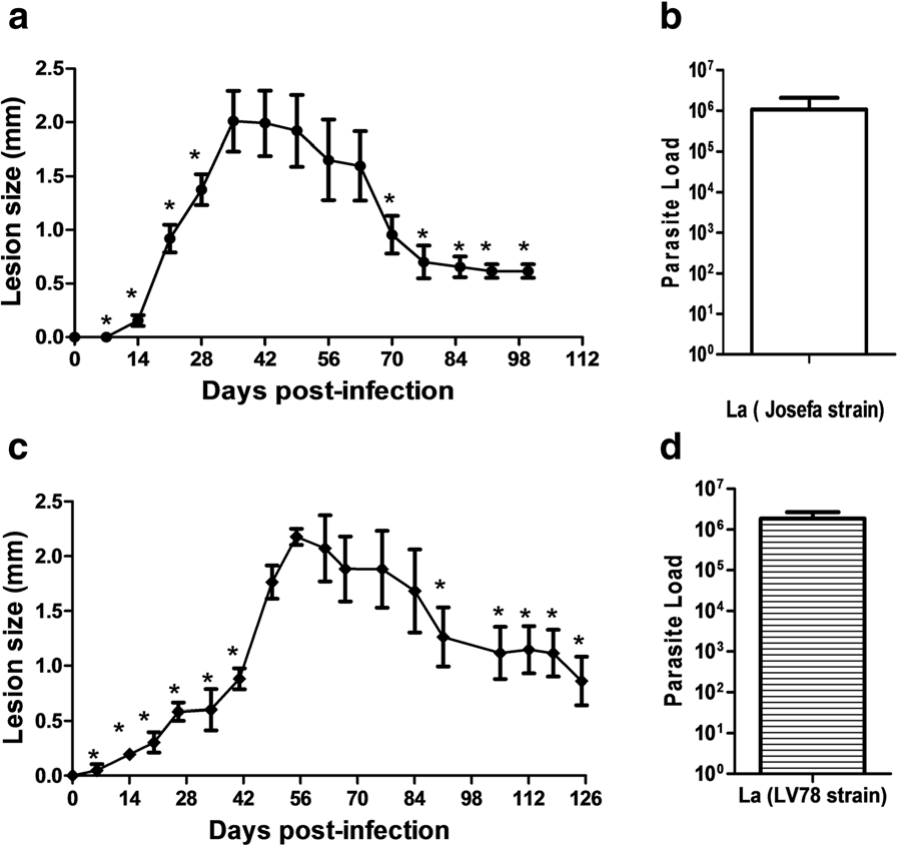
Comparison of infection of C57BL/6 mice by *L. amazonensis* Josefa strain *versus* LV78 strain. *Leishmania amazonensis* (Josefa or LV78 strains) were cultured on Schneider’s medium. C57Bl/6-UFF were infected with stationary-phase promastigotes of *L. amazonensis* Josefa strain (**a**, **b**) or LV78 strain (**c**, **d**). Lesion sizes were measured at the indicated days and expressed as the difference of thickness between non-infected and infected footpads (**a**, **c**). Parasite load was measured at the end of the experiment and expressed as the mean number of parasites per footpad (**b**, **d**). The data (means ± standard deviations; *n* = 4–5) are representative of two independent experiments producing the same result profile. *P ≤ 0.05 in comparison to peak of infection (**a**, 49 days; **c**, 55 days; see Table 2 for details)

The experiments were performed two or three times, and the result of one representative experiment is shown. For experiments illustrated in Figs. 1 and 2, differences of the peak of infection to the progressive phase and the chronic phase were tested statistically by Student's *t*-test. For the results provided in the remaining figures, differences between vaccinated and non-vaccinated groups were tested by Student’s *t*-test. We used the GraphPad Prism v. 5 software, and were considered significant when *P* ≤ 0.05.

## Results

### Characterization of the partially resistant model of *L. amazonensis* infection in C57BL/6 mice

To characterize the chronic mouse model of infection using *L. amazonensis* Josefa strain in C57BL/6 mice, we evaluated mice from different animal facilities: UNICAMP (Fig. 1a), FIOCRUZ (Fig. 1c), UFRJ (Fig. 1e) and UFF (Fig. 1g). All mice presented a similar profile after *L. amazonensis* infection, with lesion progression until days 42–60 post-infection followed by a partial resolution of the lesion, with chronic parasite persistence (Fig. 1, Table 1). Independently of the animal facility of origin, the parasite load was very similar in the chronic infection (Fig. 1b, d, f and h). The results demonstrated a partially resistant mouse model with chronic infection by *L. amazonensis*. All these experiments were performed with parasites cultured in M199 medium. To evaluate the interference of the culture medium on the infection, the assay was repeated using Schneider’s medium. Results were very similar to M199 medium, with compared lesion progression followed by partial resolution and chronic infection (Fig. 2a) and parasite load (Fig. 2b). We also evaluated this resistance model using a different strain of *L. amazonensis*, to test if this profile is general to the parasite species. Using *L. amazonensis* LV78 strain (MPRO/BR/72/M1845), we could observe a similar profile of infection (Fig. 2c, Table 2) and parasite load (Fig. 2d) in comparison to *L. amazonensis* Josefa strain. For data presented in Figs. 1 and 2, based on statistics, a lesion growth in the progressive phase, a partial lesion resolution and lesion stabilization in the chronic phase compared with the peak of infection, was observed in all experiments performed.

**Table 1:** Comparison of lesion size to size at peak of infection

	C57Bl/6-UNICAMP^a^ (Fig. 1a)			C57Bl/6-FIOCRUZ^b^ (Fig. 1c)			C57Bl/6-UFRJ^c^ (Fig. 1e)			C57BL6-UFF^d^ (Fig. 1g)	
DPI	*t*-value (*df* = 6)	*P*-value	DPI	*t*-value (*df* = 6)	*P*-value	DPI	*t*-value (*df* = 8)	*P*-value	DPI	*t*-value (*df* = 6)	*P*-value
14	20.77	<0.0001	7	15.46	<0.0001	7	12.08	<0.0001	13	7.371	0.0003
21	12.06	<0.0001	14	14.29	<0.0001	18	11.18	<0.0001	19	6.256	0.0008
28	7.997	0.0002	18	8.697	0.0001	25	10.10	<0.0001	26	4.707	0.0033
35	6.237	0.0008	25	9.102	0.0001	32	6.158	0.0003	33	3.069	0.0220
56	3.381	0.0148	39	9.133	0.0001	39	2.647	0.0294	74	3.706	0.0100
63	4.210	0.0056	46	3.245	0.0176	69	2.395	0.0435	82	3.709	0.0100
70	5.186	0.0020	68	5.106	0.0022	76	3.464	0.0085	89	3.744	0.0096
77	4.490	0.0041	80	5.178	0.0021	87	3.421	0.0091	101	3.948	0.0076
84	5.112	0.0022	89	5.213	0.0020	101	3.151	0.0136	111	3.863	0.0083
91	5.117	0.0022	96	5.404	0.0017	112	3.306	0.0108	117	3.606	0.0113
98	7.532	0.0003	104	5.126	0.0022	126	3.192	0.0128			
115	5.733	0.0012									

We evaluated mice infection with *L. amazonensis* (Josefa strain) from different animal facilities: UNICAMP, FIOCRUZ, UFRJ and UFF. The results of the Student’s t-test for lesion size from different times (days post-infection, DPI) with peak of infection are shown; see also Fig. 1

*Abbreviations*: *DPI* days post-infection, *FIOCRUZ* Fundação Oswaldo Cruz, *UFF* Universidade Federal Fluminense, *UFRJ* Universidade Federal do Rio de Janeiro, *UNICAMP* Universidade Estadual de Campinas

^a^Peak of infection at day 42 post-infection

^b^Peak of infection at day 56 post-infection

^c^Peak of infection at day 56 post-infection

^b^Peak of infection at day 53 post-infection

**Table 2:** Evaluation of lesion growth for Josefa strain and LV78 strain infections in mice

	Josefa C57Bl/6-UFF^a^ (Fig. 2a)			LV78 C57Bl/6-UFF^b^ (Fig. 2a)	
DPI	*t*-value (*df* = 8)	*P*-value	DPI	*t*-value (*df* = 8)	*P*-value
7	5.668	0.0005	6	23.16	<0.0001
14	5.233	0.0008	14	23.09	<0.0001
28	2.801	0.0232	20	15.83	<0.0001
70	2.561	0.0336	26	14.50	<0.0001
77	3.322	0.0105	34	8.164	<0.0001
85	3.644	0.0065	41	10.70	<0.0001
92	3.845	0.0049	49	2.454	0.0397
100	3.845	0.0049	91	3.274	0.0113
			105	4.253	0.0028
			112	4.512	0.0020
			118	4.712	0.0015
			125	5.632	0.0005

We evaluated mice (from UFF) infection with *L. amazonensis* using Josefa strain or LV78 strain. The results of the Student’s t-test for lesion size from different times (days post-infection) with peak of infection are shown; see also Fig. 2

*Abbreviations*: *DPI* days post-infection, *UFF* Universidade Federal Fluminense

^a^Peak of infection at day 49 post-infection

^b^Peak of infection at day 55 post-infection

To show that this infection profile was related to mice lineage and not to leishmanial strain, infection of *L. amazonensis* (Josefa strain) was performed on BALB/c mice to demonstrate a progressive (non-healing) disease in this mouse model (Additional file 1: Figure S1). The establishment and use of a partially resistant chronic infection mouse model is interesting because this model is more similar to the natural course of cutaneous infection in humans.

### Efficacy of intranasal LaAg vaccine against *L. amazonensis* infection in C57BL/6 mice

**Fig. 3:**
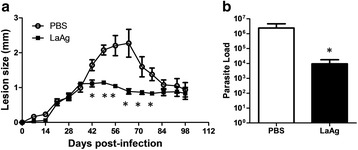
Evaluation of intranasal LaAg vaccine efficacy in the chronic stage of infection. C57Bl/6-UFF mice received 10 μg of LaAg by the intranasal route on days -14 and -7 of infection. Non-vaccinated controls received PBS alone. On day 0, animals were infected with 5 × 10^5^ promastigotes of *L. amazonensis* (Josefa strain). **a** Lesion sizes were measured at the indicated days and expressed as the difference of thickness between non-infected and infected footpads. **b** Parasite load was measured on day 98 of infection and expressed as the mean number of parasites per footpad. The data (means ± standard deviations; *n* = 4–5) are representative of three independent experiments producing the same result profile. *P ≤ 0.05 in comparison to PBS controls as follows: **a** Day 42 (*t*_(6)_ = 2.853, *P* = 0.0291); Day 49 (*t*_(6)_ = 6.113, *P* = 0.0009); Day 56 (*t*_(6)_ = 3.970, *P* = 0.0074); Day 64 (*t*_(6)_ = 3.416, *P* = 0.0142); Day 72 (*t*_(6)_ = 2.481, *P* = 0.0478); Day 78 (*t*_(6)_ = 2.921, *P* = 0,0266). **b** t_(6)_ = 3.472, *P* = 0.0070

Intranasal LaAg vaccine has been demonstrated to be effective on susceptible BALB/c mice against *L. amazonensis* infection [21]. We evaluated intranasal LaAg vaccine on C57BL/6 mice against *L. amazonensis* infection. As expected, non-vaccinated mice presented the lesion profile described above, with a progressive lesion until day 63 post-infection, when a partial lesion resolution ensued associated with a chronic resistant lesion (Fig. 3a). Immunized mice controlled the lesion progression from day 42 post-infection (Fig. 3a). After partial lesion resolution, both, PBS and LaAg, showed the same lesion size after day 84 post-infection (Fig. 3a). However, the parasite load at day 98 day post-infection demonstrated that intranasal LaAg vaccine reduced the number of parasites in the chronic infection (Fig. 3b).

**Fig. 4:**
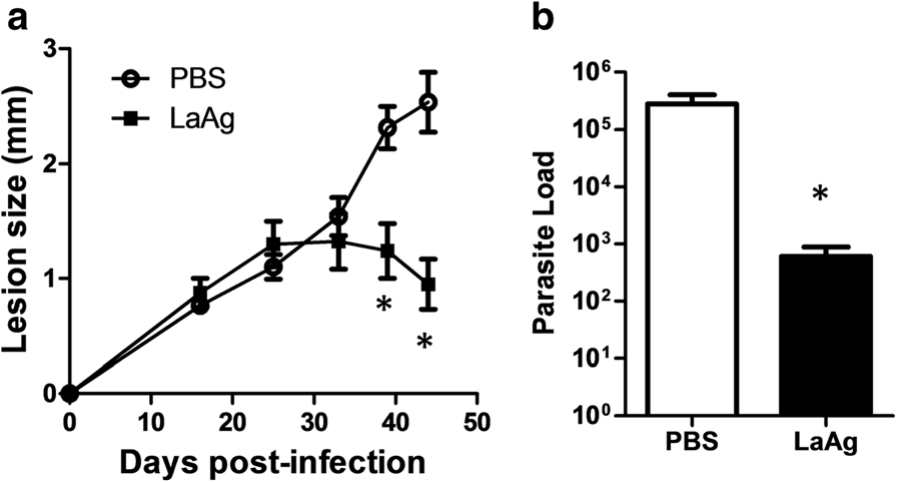
Evaluation of intranasal LaAg efficacy in the progressive stage of infection. C57Bl/6-UFF mice received 10 μg of LaAg by the intranasal route on days -14 and -7 of infection. Non-vaccinated controls received PBS alone. On day 0, animals were infected with 5 × 10^5^ promastigotes of *L. amazonensis* (Josefa strain). **a** Lesion sizes were measured at the indicated days and expressed as the difference of thickness between non-infected and infected footpads. **b** Parasite load was measured on day 44 of infection and expressed as the mean number of parasites. The data (means ± standard deviations; *n* = 5) are representative of three independent experiments producing the same result profile. *P* ≤ 0.01 in comparison to PBS controls as follows: **a** Day 39 (*t*_(7)_ = 3.566, *P* = 0.0073); Day 44 (*t*_(7)_ = 5.037, *P* = 0.0015). **b** *t*_(7)_ = 4.614, *P* = 0.0024

Varying the number of parasites used to infect mice, low model of infection (challenge with 5 × 10^5^ parasites) and high model of infection (challenge with 2 × 10^6^ parasites), we observed the same profile of lesion progression control (Additional file 2: Figure S2a) and reduction of parasite load (Additional file 2: Figure S2b) following LaAg vaccination. To determine the parasite load during lesion progression, we vaccinated mice and evaluated lesion progression and parasite load at day 44 post-infection. As expected, we could observe the control of lesion progression (Fig. 4a) and a reduction in parasite load, showing that parasite control happens in parallel to lesion progression inhibition (Fig. 3) in vaccinated mice.

### Intranasal LaAg vaccine induced a Th1 response

**Fig. 5:**
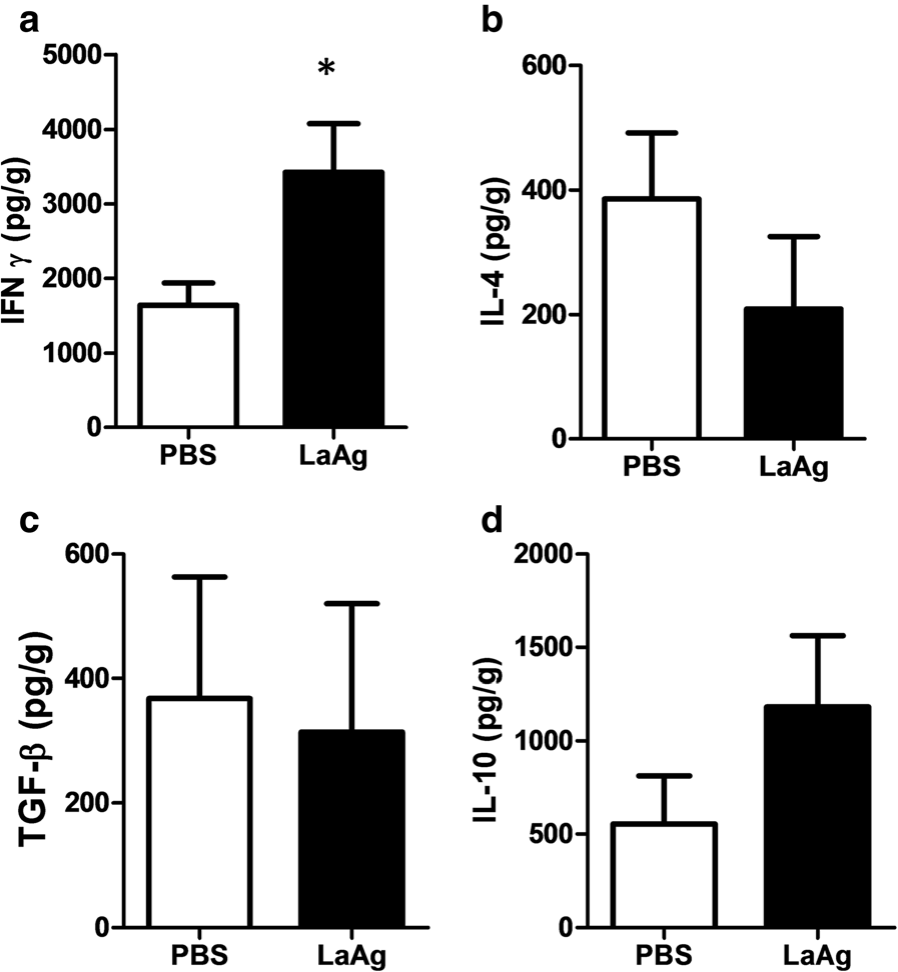
*In situ* cytokine profile in the acute stage of infection. C57Bl/6 mice (from UFF) received 10 μg of LaAg by the intranasal route on days -14 and -7 of infection. Non-vaccinated controls received PBS alone. On day 0, animals were infected with 5 × 10^5^ promastigotes of *L. amazonensis* (Josefa strain). On day 44 of infection (see Fig. 4), the levels of IFN-γ (**a**), IL-4 (**b**), TGF-β (**C**), IL-10 (**d**) were measured in the lesion homogenates. The data (means ± standard deviations; *n* = 4–5) are representative of two independent experiments. *P ≤ 0.05 in comparison to PBS controls (t_(6)_ = 2.491, *P* = 0.0471)

To evaluate the mechanism of vaccine protection, we quantified *in situ* cytokine levels in the footpad homogenates. We could observe during the lesion progression at day 44 post-infection that LaAg induced in vaccinated mice an increase in IFN-gamma release (Fig. 5a) that paralleled the lesion control (Fig. 4a) and reduction in parasite load (Fig. 4b). However, no modulation of IL-4 (Fig. 5b), TGF-beta (Fig. 5c) and IL-10 (Fig. 5d) were detected. In the chronic infection at day 98 post-infection, despite the reduction in parasite load (Fig. 3b), we could not detect any modulation of IFN-gamma (Additional file 3: Figure S3a), IL-4 (Additional file 3: Figure S3b), TGF-beta (Additional file 3: Figure S3d) and IL-10 (Additional file 3: Figure S3c). Probably, the immune modulation during the lesion progression was enough to decrease and maintain a reduced parasite load, and it is important to point out that the level of IFN-gamma is higher in the chronic phase in comparison to the progressive phase, probably associated to the self-healing (lesion resolution) process. In a preliminary experiment, we observed, in the peak of infection at 44 days post-infection, an induction of CD4^+^ IFN-γ^+^ T cells by intranasal LaAg vaccine in comparison to PBS (Additional file 4: Figure S4f) in popliteal lymph node cells. We could not detect any difference in CD8^+^ IFN-γ^+^ T cells at the peak of infection (result not shown). This result suggests CD4^+^ T cells as the major mechanism of Th1 response by Intranasal LaAg vaccine.

### Association of LaAg with Addavax® adjuvant did not enhance the protective efficacy

**Fig. 6:**
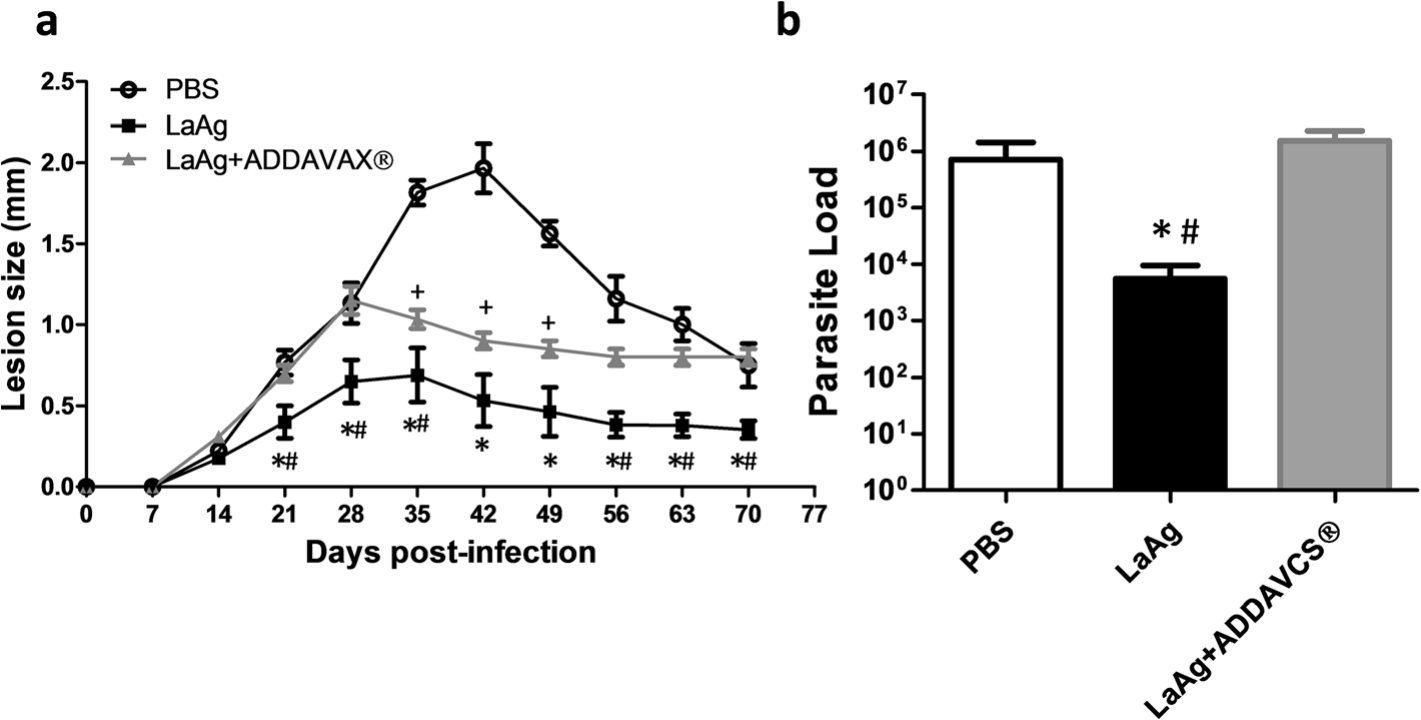
Evaluation of intranasal LaAg vaccine associated with ADDAVAX®. C57Bl/6-UFF mice received 10 μg of LaAg (10 μl) associated or not with ADDAVAX (10 μl) by the intranasal route on days -14 and -7 of infection. Non-vaccinated controls received PBS alone. On day 0, animals were infected with 5 × 10^5^ promastigotes of *L. amazonensis* (Josefa strain). **a** Lesion sizes were measured at the indicated days and expressed as the difference of thickness between non-infected and infected footpads. **b** Parasite load was measured on day 70 of infection and expressed as the mean number of parasites in each footpad. The data (means ± standard deviations; *n* = 5–6) are representative of three independent experiments producing the same result profile. *P ≤ 0.05: LaAg in comparison to PBS controls; ^#^ *P* ≤ 0.05: **b** LaAg in comparison to LaAg + ADDAVAX; +*P* ≤ 0.05; LaAg + ADDVACS in comparison to PBS controls Test statistics for **a** are provided in Table 3. **b** LaAg in comparison to PBS: *t*_(8)_ = 5.788, *P* = 0.0022; LaAg in comparison to LaAg + Addavacs: (*t*_(8)_ = 6.501, *P* = 0.0013)

Scalene based adjuvant known as MF59 was the first approved adjuvant to be used by intranasal route in the Flu vaccine [32]. Addavax® is a nano emulsion based on scalane oil-water emulsion from Invitrogen. Based on the capacity to induce T cell response by intranasal route of scalene- based adjuvants [32], we hypothesized the association of LaAg with Addavax® could improve the vaccine efficacy. Surprisingly, the association of LaAg with Addavax® partially impaired the lesion control promoted by LaAg (Fig. 6a, Table 3) and reverted its parasite load control in chronic infection (Fig. 6b). The administration of Addavax® alone by intranasal route did not affect the lesion and parasite load (data not shown).

**Table 3:** Evaluation of LaAg vaccine associated or not with ADDAVACS®

	LaAg *vs* PBS		LaAg *vs* LaAg + ADDAVACS		LaAg + ADDAVACS *vs* PBS	
DPI	*t*-value (*df* = 8)	*P*-value	*t*-value (*df* = 8)	*P*-value	*t*-value (*df* = 8)	*P*-value
21	7.117	0.0001	6.424	0.0002	–	–
28	7.363	<0.0001	8.113	<0.0001	–	–
35	16.86	<0.0001	5.000	0.0011	20.68	<0.0001
42	16.68	<0.0001	–	–	16.71	<0.0001
49	16.58	<0.0001	–	–	18.65	<0.0001
56	14.28	<0.0001	12.36	<0.0001	–	–
63	13.07	<0.0001	13.46	<0.0001	–	–
75	8.180	<0.0001	15.91	<0.0001	–	–

. Mice were vaccinated twice with LaAg or LaAg plus ADDAVACS or control (PBS) and then mice were infected. The results of the Student’s t-test for lesion size at different days post-infection between experimental groups (LaAg *versus* PBS; LaAg *versus* LaAg plus ADDAVACS; and LaAg plus ADDAVACS *versus* PBS) are shown; see also Fig. 6a

*Abbreviation*: *DPI* days post-infection

## Discussion

Before clinical studies for vaccines, it is necessary to perform very robust pre-clinical studies using different infection models, such as mice, dog and non-human primates [36]. Intranasal LaAg vaccine is protective to BALB/c mice against *L. amazonensis* [21] and *L. infantum*/*chagasi* infection [23] and to hamsters against *L. braziliensis* [29]. LaAg ability to protect against different parasite species (*L. amazonensis*, *L. chagasi* and *L. braziliensis*) and positive results in two different species (BALB/c and Hamster) is very promising. However, it is very important to find the best model to evaluate LaAg vaccine efficacy [37]. In this study, we evaluated immunization against *L. amazonensis* infection in the C57BL/6 mouse model, which displays a different profile of infection.

At the beginning, we characterized the infection of *L. amazonensis* using Josefa strain on C57BL/6 mice. In the early infection, infected mice presented a progressive phase (42–60 days post-infection), followed by a partial resolution and chronic infection (Figs. 1 and 2). Human cutaneous leishmaniasis infection is a self-healing disease, however, parasites can be found in healed lesions [38]. Human disease is very different from the clinical outcome observed in BALB/c mice [12], being more alike to C57BL/6 mice described here. Although BALB/c mice have been used for drug trials, it is necessary to use a self-healing model that more closely reproduces the natural infection course in humans to evaluate and confirm the efficacy of these compounds [39]. The same concept has to be transposed for vaccine development. It is important that differences between experimental models and humans are accounted for in vaccine development [40]. We presented here a partially resistant mouse model using C57BL/6 mice with a chronic infection with persistent parasite load. Using this model it is possible to evaluate the efficacy of LaAg vaccine in the progressive phase (Fig. 4) and in the chronic phase (Fig. 3). In vaccinated mice, the control of lesion growth (Figs. 3a and 4a) is very important to avoid tissue destruction. The partial reduction of parasite load (Figs. 3b and 4b) could also be important to prevent disease transmission in the progressive phase and in the chronic phase [41].

For standardization of our mouse model, we evaluated mice from different animal facilities and parasites grown in different culture medium. It has previously been described that mice from different facilities could present different microbiota, and this can influence their immune response [14, 42]. We used C57BL/6 mice originally from Jackson Laboratories, however, housed and bred in UNICAMP, FIOCRUZ, UFRJ and UFF animal facilities. Our experiments demonstrated that independent of facility, the infection profiles were very similar (Fig. 1). These results minimize the possibility that results are relevant only for animals from a specific supplier.

Then, we tested different culture media for *Leishmania* growth and infectivity. The three more important media (199 medium, Grace’s insect tissue-culture medium and Schneider’s Drosophila Medium) have been used for a long time [43]. In this study, we evaluated *L. amazonensis* infectivity after growth in 199 (Fig. 1) and Schneider’s (Fig. 2) medium, and no difference was observed on the profile of infection. Besides, we evaluated different numbers of parasites used to infect mice: 2 × 10^5^ and 2 × 10^6^. There was no difference in the profile either (data not shown).

It is important to note that different strains of the same parasite can present different disease progression, for example, for *Leishmania major*, the strain V1 (MHOM/IL/80/Friedlin) has a healing model, but the strain Sd (MHOM/SN/74/SD) is a progressive non-healing model in C57BL/6 mice [11]. There are three *L. amazonensis* strains being used for research in Brazil: Josefa strain (used in this work), PH8 and LBT0016. LBT0016 was isolated from cutaneous leishmaniasis; Josefa strain was also isolated from cutaneous leishmaniasis [33] and not from diffuse cutaneous leishmaniasis [44]. Thus, this strain was isolated from a patient with the most prevalent presentation of the disease and reproduced the same infection profile after inoculation in mice. LV78 (results herein) and LBT0016 strains also showed the same profile of infection, and as such, are an interesting model to evaluate the impact of vaccines relevant to human leishmaniasis.

However, *L amazonensis* (MHOM/BR/76/Ma-5) isolated from a human patient with cutaneous diffuse leishmaniasis demonstrated a different profile, presenting a progressive lesion on C57BL/6 mice until 90 days post-infection [45]. In the chronic phase, despite the presence of a large lesion, it was not possible to detect parasites [45]. Others demonstrated that intradermal infection on ears of C57BL/6 mice using *L amazonensis* PH8 strain, isolated from sand flies, showed a progressive disease with a chronic lesion, in other words, in the chronic phase, the lesion was not uncontrolled; however, also did not heal [46, 47]. The different site of infection (ear) or the different route of infection (intradermal) from subcutaneous injection in the hind paw could affect the lesion progression [12]. These results demonstrate that each parasite should be empirically evaluated to determine the behaviour of infection in mice, but they seem to generally reproduce in the animal model the original behaviour in lesions of human patients. The model used herein presents a chronic phase with a high parasite load resembling the natural history of leishmaniasis and is more interesting for vaccine evaluation due to this similarity with human disease outcome (progressive phase, partial resolution and chronic phase development).

*Leishmania amazonensis* has the capacity to induce a mixed cytokine response, Th1-IFN-gamma/Th2-IL-4 [48], IL-10 [49] and TGF-beta [20, 50]. Immunization did not modulate IL-4, IL-10 or TGF-beta, maybe indicating a secondary role of these molecules in a vaccine context. The protection observed by intranasal LaAg vaccine on C57BL/6 mice was correlated to IFN-gamma levels in the lesions (Fig. 5). IFN-gamma is a crucial cytokine to control *L. major* [51, 52] and *L. donovani* infection [53]. IFN-gamma is described to increase *L. amazonensis* parasite load in vitro [54], however, in vivo it is considered important for infection control [55]. Moreover, production of IFN-gamma in the site of infection in BALB/c mice is associated with protection against *L. amazonensis* infection [24]. The mechanism of intranasal LaAg vaccine against *L. amazonensis* in BALB/c [21]; *L. chagasi* in BALB/c [26]; *L. braziliensis* in hamster [29]; and now *L. amazonensis* in C57BL/6 mice, is associated with IFN-gamma production. These results together demonstrate the importance of IFN-gamma as the major marker for vaccine studies against leishmaniasis. In preliminary experiments, we suggested the participation of CD4+ T cells to produce Interferon-gamma (Additional file 4: Figure S4f) in LaAg vaccine, as indicated for several studies as the most important Th1 parasitic-specific response against leishmaniasis [56].

The human vaccine candidate has to be feasible to protect against different parasites and against different clinical forms [56–58]. Intranasal LaAg vaccine has demonstrated being effective in different mouse models, against different *Leishmania* species and with different forms of disease [21, 26, 29]. In our work, the choice of a model of infection more similar to human infection based on the self-healing in human with normal immunity using C57BL/6 mice allowed us to do consideration about the LaAg vaccine. The efficacy of the vaccine in the control of the lesion size in the progressive phase is very interesting. Besides, there is a reduction of parasite load in the chronic phase in mice, demonstrating the quality of this vaccine. When we considered the efficacy on BALB/c mice, we can transpose the vaccine against the severe form of disease to cutaneous diffuse leishmaniasis based on the uncontrolled parasite load. The perspective of LaAg intranasal vaccine as a human vaccine candidate is due its capacity to reduce the size of the lesion and control the parasite load. Intranasal LaAg vaccine has all the concepts expected for a human vaccine candidate.

The importance of adjuvants to enhance the immune response of vaccines is already known, and new adjuvants based on squalene emulsion open the possibility to development of new vaccines [32]. The association with ADDAVAX® adjuvant can enhance the protection in some vaccines [59], and hinders efficacy for others [60]. This type of adjuvant has been used to enhance both Th1 and Th2 responses [61, 62]. Here, we demonstrated that the use of LaAg associated to ADDAVAX® decreased the LaAg vaccine efficacy (Fig. 6). The protection of LaAg adjuvant free is very encouraging, but we are still looking for new adjuvants to enhance LaAg protection [26] and for characterization of LaAg components to developing more defined vaccines [24, 63–65] .

## Conclusion

Adjuvant free LaAg by intranasal route is protective against *L. amazonensis* infection using the C57BL/6 mouse model that more closely reproduces the infection profile in humans. The efficacy against other parasites such as *L. chagasi* and *L. braziliensis* point to intranasal LaAg immunization as a promising vaccine candidate against leishmaniasis.

## Additional files

Additional file 1: Figure S1. Course of infection by *L. amazonensis* in BALB/c mice. BALB/c mice were infected with 5 × 10^5^ stationary-phase promastigotes of *L. amazonensis* (Josefa strain) in the footpads. **a** Lesion sizes were measured at the indicated days and are expressed as the difference of thickness between non-infected and infected footpads. **b** Parasite load was measured in the chronic infection phase and expressed as mean number of parasites. The data (means ± standard deviations; *n* = 5) are representative of three independent experiments producing the same result profile. (TIF 210 kb)
Additional file 2: Figure S2. Efficacy of intranasal LaAg against high challenge of *L. amazonensis* infection on C57BL/6. C57Bl/6-UFF received 10 μg of LaAg by the intranasal route on days -14 and -7 of infection. Non-vaccinated controls received PBS alone. On day 0, animals were infected with 2 × 10^6^ stationary-phase promastigotes of *L. amazonensis* (7 days of culture). a Lesion sizes were measured at the indicated days and are expressed as the difference of thickness between non-infected and infected footpads. b The parasite load was measured on day 98 of infection and is expressed as number of parasites. The data (means ± standard deviations; *n* = 4–5) are representative of three independent experiments producing the same result profile. **P* ≤ 0.05 in comparison to PBS controls. **a** Day 38 (t_(6)_ = 3.303, *P* = 0.0164), Day 45 (t_(6)_ = 2.813, *P* = 0.0306), Day (t_(6)_ = 3.743, *P* = 0.0096), Day 61 (t_(6)_ = 6.917, *P* = 0.0010); Day 70 (t_(6)_ = 3.757, *P* = 0.0198). **b** t_(6)_ = 6.778, *P* = 0.0025. (TIF 232 kb)
Additional file 3: Figure S3. In situ cytokine profile in the chronic phase. Mice (from UFF) were intranasally vaccinated with LaAg and infected with *L. amazonensis* as described for data in Fig. 3. On day 110 of infection (see Fig. 3), the levels of IFN-γ (**a**), IL-4 (**b**), TGF-β (**c**), IL-10 (**d**) were measured in the lesion homogenates, and expressed as ng per g of footpad. The data (means ± standard deviations; *n* = 4–5) are representative of three independent experiments. **P* ≤ 0.05 in comparison to PBS controls. (TIF 285 kb)
Additional file 4: Figure S4. LaAg vaccine induced CD4^+^ IFN-γ^+^ T cells in progressive stage of infection. Mice were vaccinated and infected as in the experiment in Fig. 4. Lymph nodes were removed at day 44 post-infection. Frequency CD3^+^CD4^+^ cells from PBS mice (**a**) and from LaAg-vaccinated mice (**b**). Dot plot (frequency) of IFN-γ staining of CD4^+^ from PBS mice (**c**) or LaAg-vaccinated mice (D). **e** Number of CD3^+^CD4^+^ cells for each mouse from PBS and LaAg-vaccinated groups. **f** The production of IFN-γ^+^ by CD3^+^CD4^+^ cells was calculated for each mouse. The data (means ± standard deviations; *n* = 4–6) are from one experiment. **P* ≤ 0.01 in comparison to PBS controls (cf. Fig. 4f, t_(10)_ = 3.410, *P* = 0.0067). (TIF 4245 kb)

## Abbreviations

LaAg: *Leishmania amazonensis* antigens
LDA: Limited dilution assay
DPI: Days post-infection
