# Supplementary figures and images for: Efficacy of intranasal LaAg vaccine against Leishmania amazonensis infection in partially resistant C57Bl/6 mice
Source: Parasit Vectors. 2016 Oct 6;9:534. doi: 10.1186/s13071-016-1822-9 (PMC5052793; doi:10.1186/s13071-016-1822-9)

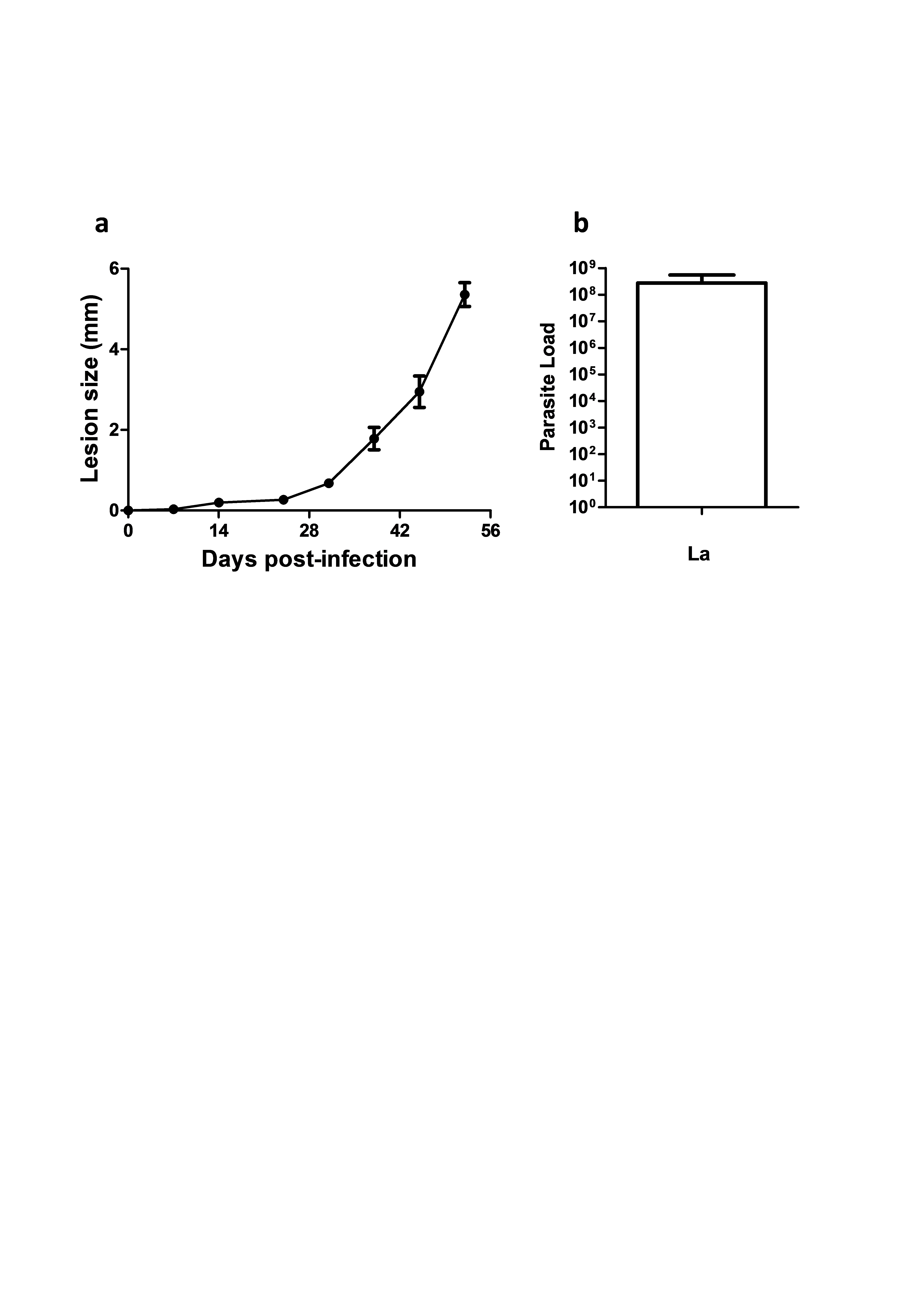

Supplement: Additional file 1: Figure S1. — Course of infection by L. amazonensis in BALB/c mice. BALB/c mice were infected with 5 × 10^5^ stationary-phase promastigotes of L. amazonensis (Josefa strain) in the footpads. a Lesion sizes were measured at the indicated days and are expressed as the difference of thickness between non-infected and infected footpads. b Parasite load was measured in the chronic infection phase and expressed as mean number of parasites. The data (means ± standard deviations; n = 5) are representative of three independent experiments producing the same result profile. (TIF 210 kb) [file 13071_2016_1822_MOESM1_ESM.tif]

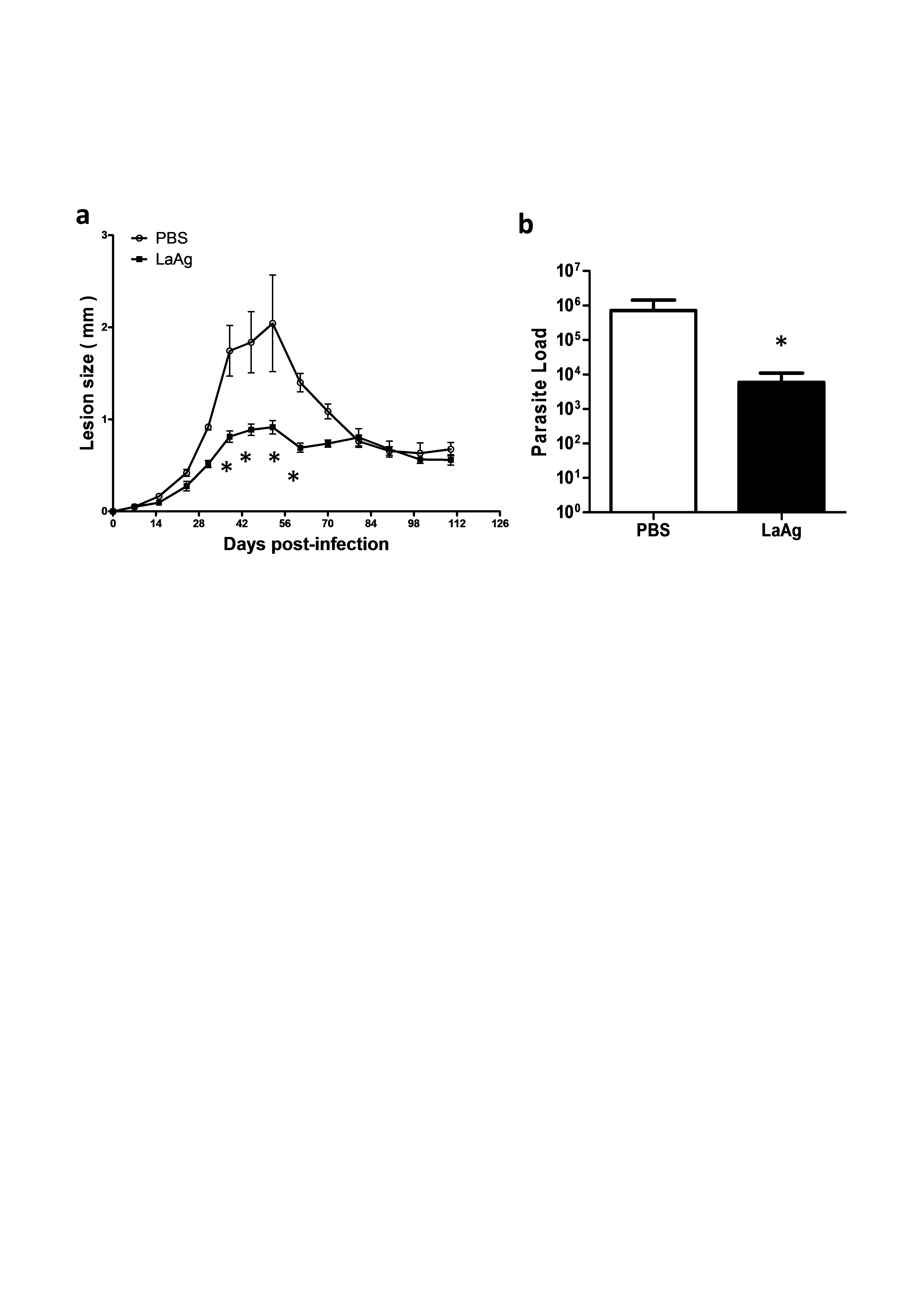

Supplement: Additional file 2: Figure S2. — Efficacy of intranasal LaAg against high challenge of L. amazonensis infection on C57BL/6. C57Bl/6-UFF received 10 μg of LaAg by the intranasal route on days -14 and -7 of infection. Non-vaccinated controls received PBS alone. On day 0, animals were infected with 2 × 10^6^ stationary-phase promastigotes of L. amazonensis (7 days of culture). a Lesion sizes were measured at the indicated days and are expressed as the difference of thickness between non-infected and infected footpads. b The parasite load was measured on day 98 of infection and is expressed as number of parasites. The data (means ± standard deviations; n = 4–5) are representative of three independent experiments producing the same result profile. *P ≤ 0.05 in comparison to PBS controls. a Day 38 (t(6) = 3.303, P = 0.0164), Day 45 (t(6) = 2.813, P = 0.0306), Day (t(6) = 3.743, P = 0.0096), Day 61 (t(6) = 6.917, P = 0.0010); Day 70 (t(6) = 3.757, P = 0.0198). b t(6) = 6.778, P = 0.0025. (TIF 232 kb) [file 13071_2016_1822_MOESM2_ESM.tif]

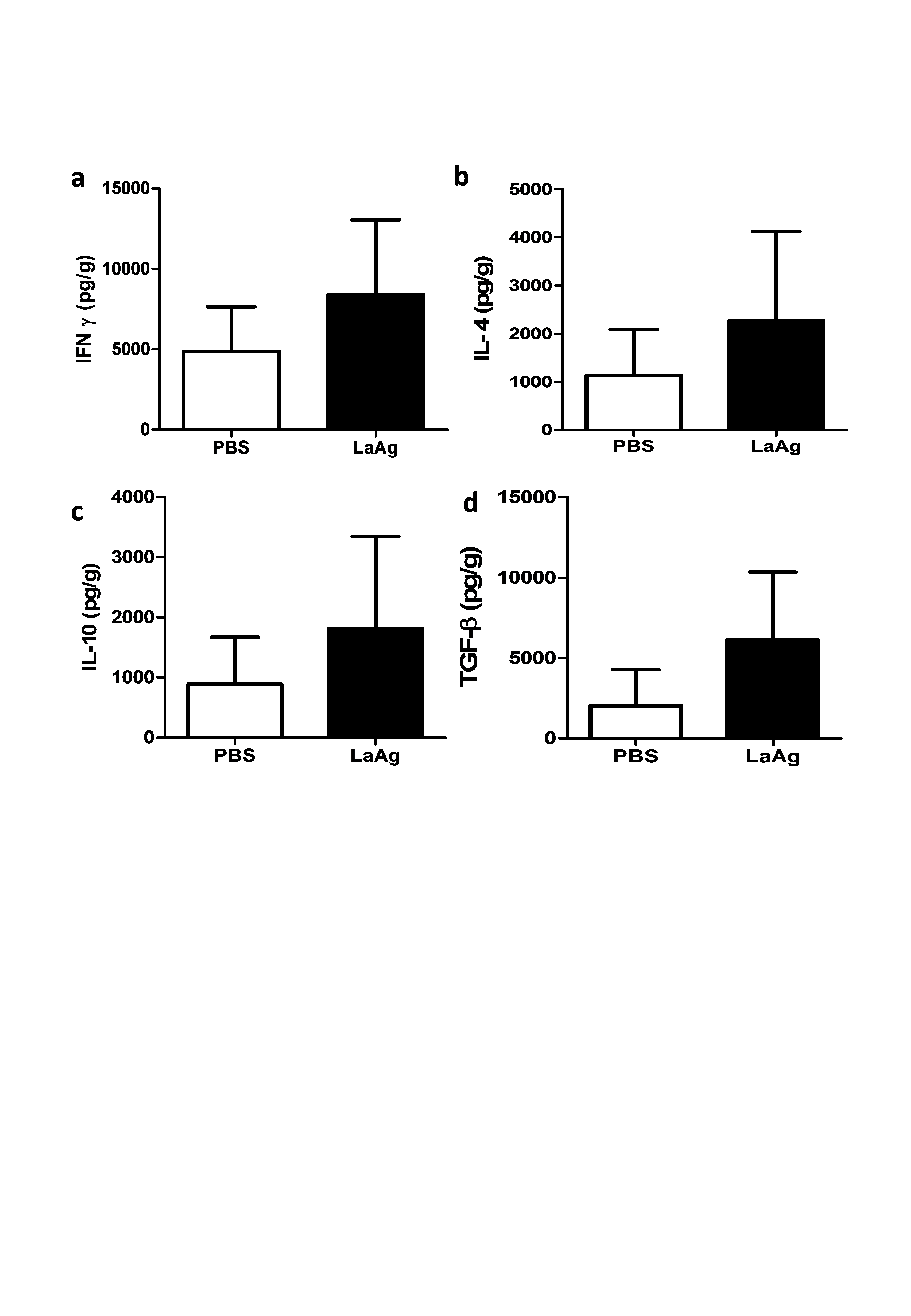

Supplement: Additional file 3: Figure S3. — In situ cytokine profile in the chronic phase. Mice (from UFF) were intranasally vaccinated with LaAg and infected with L. amazonensis as described for data in Fig. 3. On day 110 of infection (see Fig. 3), the levels of IFN-γ (a), IL-4 (b), TGF-β (c), IL-10 (d) were measured in the lesion homogenates, and expressed as ng per g of footpad. The data (means ± standard deviations; n = 4–5) are representative of three independent experiments. *P ≤ 0.05 in comparison to PBS controls. (TIF 285 kb) [file 13071_2016_1822_MOESM3_ESM.tif]

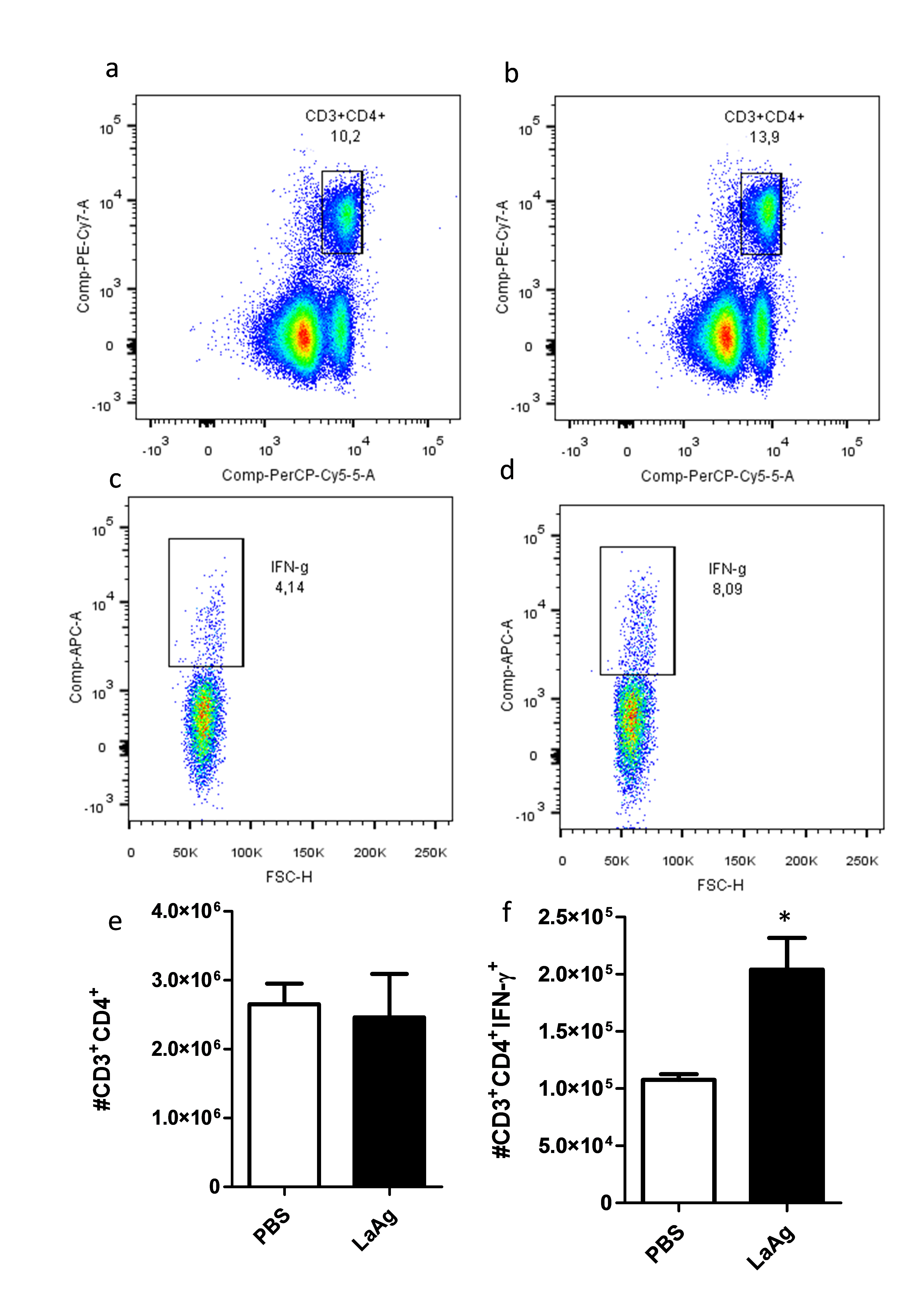

Supplement: Additional file 4: Figure S4. — LaAg vaccine induced CD4+ IFN-γ+ T cells in progressive stage of infection. Mice were vaccinated and infected as in the experiment in Fig. 4. Lymph nodes were removed at day 44 post-infection. Frequency CD3+CD4+ cells from PBS mice (a) and from LaAg-vaccinated mice (b). Dot plot (frequency) of IFN-γ staining of CD4+ from PBS mice (c) or LaAg-vaccinated mice (D). e Number of CD3+CD4+ cells for each mouse from PBS and LaAg-vaccinated groups. f The production of IFN-γ+ by CD3+CD4+ cells was calculated for each mouse. The data (means ± standard deviations; n = 4–6) are from one experiment. *P ≤ 0.01 in comparison to PBS controls (cf. Fig. 4f, t (10) = 3.410, P = 0.0067). (TIF 4245 kb) [file 13071_2016_1822_MOESM4_ESM.tif]
